# Correction to ‘Moving conferences online: lessons learned from an international virtual meeting’

**DOI:** 10.1098/rspb.2022.0321

**Published:** 2022-03-30

**Authors:** Paris V. Stefanoudis, Leann M. Biancani, Sergio Cambronero-Solano, Malcolm R. Clark, Jonathan T. Copley, Erin Easton, Franziska Elmer, Steven H. D. Haddock, Santiago Herrera, Ilysa S. Iglesias, Andrea M. Quattrini, Julia Sigwart, Chris Yesson, Adrian G. Glover

*Proc. R. Soc. B* **288**, 20211769 (Published 20 October 2021). (doi:10.1098/rspb.2021.1769)

In figure 3*e* of our original paper, the middle pie chart had the colour schemes for the different career stage groups mixed up. In that paper, 17% was dark blue, 31% was blue and 52% was light blue. It should be 17% blue, 31% light blue and 52% dark blue, as shown in the corrected version below.

**Figure 3.**
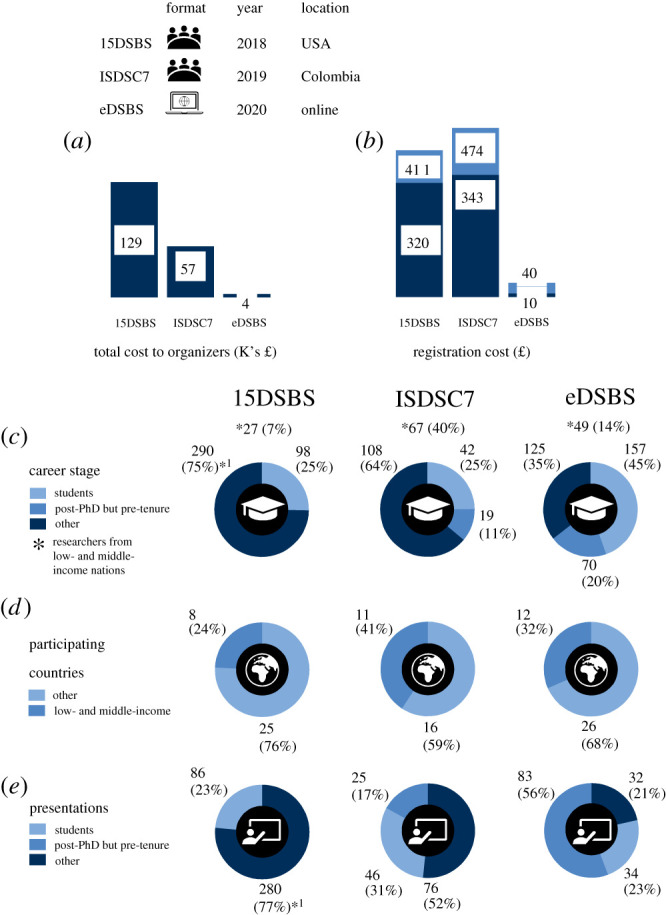
Comparison between similar-themed online and in-person meetings. (*a*) Total cost to organizers, using currency conversion rates as of 1 March 2021. Conversions were rounded to the nearest integer*.* (*b*) Registration cost to participants, indicating reduced (dark blue) and standard (light blue) registration fee options, linked to career stage and country of institutional affiliation. (*c*) Demographic composition by career stage. Note that students include PhD candidates too, while tenure includes any equivalent permanent position. (*d*) Number of participating countries, as identified from participants' institutional affiliations. Country categories based on the 2021 classification by the World Bank (last accessed on 19 January 2021). (*e*) Presentation composition by career stage. ^*1^Includes post-PhD but pre-tenure. (Online version in colour.)

